# Incidence and risk factors for catheter-related thrombosis in elderly patients with malignant tumors: a single-center retrospective cohort study

**DOI:** 10.3389/fonc.2026.1843941

**Published:** 2026-08-28

**Authors:** Yanli Wang, Xiaoli Mei, He Liu, Fuqiu Tang

**Affiliations:** 1 Department of Obstetrics and Gynecology, General Hospital of Central Theater Command, People’s Liberation Army, Wuhan, Hubei, China; 2 Department of Gastroenterology, General Hospital of Central Theater Command, People’s Liberation Army, Wuhan, Hubei, China; 3 Department of Nuclear Medicine, General Hospital of Central Theater Command, People’s Liberation Army, Wuhan, Hubei, China; 4 Cadre Ward Two, General Hospital of Central Theater Command, People’s Liberation Army, Wuhan, Hubei, China

**Keywords:** cancer, catheter-related thrombosis, elderly patients, prediction model, thrombosis

## Abstract

**Background:**

Peripherally inserted central catheters (PICCs) are widely used in hospitalized patients with cancer for chemotherapy, nutritional support, and routine medication infusion. Catheter-related venous thrombosis (CRT) is a severe complication that is associated with poor prognosis, especially among elderly patients with cancer. However, studies on the identification of risk factors for CRT in elderly patients with cancer are limited.

**Methods:**

From June 2024 to May 2025, clinical data from hospitalized elderly patients (aged over 60 years) with digestive system tumors (DSTs) or gynecologic tumors (GTs) were collected. A total of 270 patients who underwent PICC were enrolled. Among them, 46 patients were diagnosed with CRT. Basic demographic data, clinical records, catheter placement details, and coagulation indicators were collected. The risk factors for CRT were assessed via univariate and multivariate unconditional logistic regression. Receiver operating characteristic (ROC) curve analysis was subsequently performed to verify the efficacy of combined markers for predicting CRT.

**Results:**

There were 120 patients with DSTs and 150 patients with GTs. Symptomatic CRT occurred in 46 patients (17.04%), including 26 (56.5%) with DSTs and 20 (43.5%) with GTs. Compared with the non-CRT group, white blood cell (WBC) count and D-dimer and hs-CRP levels were significantly increased in the CRT group, whereas the platelet count was significantly decreased. Multivariate regression analysis revealed that a history of thromboembolism, use of mechanical ventilation, distant metastasis of cancer, elevated WBC count, D-dimer, hs-CRP, and decreased platelet count were independent risk factors for CRT in hospitalized patients with tumors (P < 0.05). A predictive nomogram incorporating these four variables was constructed. The model demonstrated excellent discrimination, with an apparent C-statistic of 0.992 in the training cohort, an optimism-corrected AUC of 0.986, and an AUC of 0.971 (95% CI: 0.93–1.00) in the independent test set.

**Conclusion:**

Elevated WBC counts, D-dimer and hs-CRP levels, and decreased platelet counts are independent risk factors for CRT among hospitalized elderly patients with cancer. The predictive model shows promising discriminative ability and potential clinical utility, warranting further external validation.

## 1 Introduction

Central venous catheters (CVCs) are the primary infusion devices used for chemotherapy in cancer patients and can be worn long term, ranging from several months to several years (1). In the United States, at least 150 million to 200 million venous catheters are placed annually, with up to 80% of hospitalized patients receiving intravenous infusions (2). These plastic tubes deliver medications, nutrients, or blood directly into veins and reduce the risk of peripheral vascular irritation (3). In terms of the insertion route and implantation method, CVCs can be classified into four main types: nontunneled central venous catheters, tunneled central venous catheters, peripherally inserted central catheters (PICCs), and totally implanted venous access ports (TIVAPs) (4). Prolonged hospitalization and improper catheter maintenance can lead to catheter-related bloodstream infection (CRBSI), catheter-related thrombosis (CRT), and catheter-related local complications (CRLCs).

Cancer patients face a higher risk of CRT. A retrospective analysis revealed that the incidence of CRT in cancer patients is approximately 35% (5), and the incidence of TIVAPs is lower than that of PICCs (6). CRT may have negative impacts on the prognosis of patients receiving antitumor therapy by causing venous access obstruction, leading to delays or interruptions in chemotherapy, and may also result in fatal consequences such as pulmonary embolism and death (7). The pathogenesis of CRT shares the same mechanisms as systemic thrombosis, which include endothelial injury, hypercoagulability, and abnormal blood flow. Thrombus formation arises from the interplay of three key mechanisms: first, direct mechanical damage to the vascular endothelium during catheter placement; second, hemodynamic alterations—including diminished flow velocity and turbulent vortex development—stemming from the catheter itself or the administration of irritating substances such as chemotherapeutic or hyperosmolar agents; and third, a prothrombotic state driven by underlying conditions such as malignancy, sepsis, or localized inflammatory responses (8, 9). Risk factors associated with CRT can be divided into two major categories: treatment-related factors and catheter-related factors during treatment. Treatment-related factors include tumor burden, cancer treatment history, prior CRT events, clinical status, and baseline thrombotic risk. Catheter-related factors include the CVC type, indwelling duration, insertion site, and number of lumens (6, 10). Typical symptoms of CRT include pain, swelling, or catheter obstruction. Clinically, CRT is often confirmed by venography or vascular ultrasound, which can identify the presence of occlusive or nonocclusive thrombosis within the vessel (11). However, owing to the lack of obvious clinical manifestations, asymptomatic arterial thrombosis is often missed or undiagnosed because it is not detected promptly.

Currently, the treatment goals for CRT are to improve acute thrombotic symptoms, restore CVC patency, promote thrombus recanalization, and prevent recurrent thromboembolic events. Owing to the increased safety and convenience of low-molecular-weight heparins (LMWHs), such as enoxaparin or fondaparinux, they are generally recommended over unfractionated heparin (12). Multiple studies have shown that direct oral anticoagulants (DOACs) perform similarly to LMWHs in terms of recurrence rate, bleeding rate, and mortality and have been accepted for CRT treatment (13, 14). Although the duration of anticoagulant therapy is still controversial, it often lasts for over 3 months (15). Moreover, the treatment plan needs to follow individualized principles, requiring a comprehensive assessment of the patient’s thrombotic risk and bleeding risk (16).

This study retrospectively investigated the incidence and characteristics of CRT in elderly patients with malignancies. Potential risk factors influencing CRT were assessed through multivariate logistic regression analysis. The aim is to establish a theoretical foundation for the prevention and management of CRT in this susceptible population.

## 2 Methods

### 2.1 Study population

This retrospective cohort study included patients with malignant tumors who were admitted to the General Hospital of Central Theater Command People’s Liberation Army between June 2024 and May 2025 and who underwent PICC placement during hospitalization. We adhered to the reporting standards set by the “Observational Study Report” and regularly collected health data in this study (17). This study was approved by the Human Clinical Research Ethics Committee of the General Hospital of Central Theater Command People’s Liberation Army (L-202402-36). Owing to the retrospective and observational nature of the study, informed consent was not obtained. This research was conducted in accordance with the principles of the Declaration of Helsinki.

The inclusion criteria were as follows: (1) pathologically confirmed digestive system tumors (DSTs) or gynecologic tumors (GTs); (2) age >60 years; (3) meeting the conditions for regular catheter maintenance and indications for catheter retention; and (4) ability to tolerate color Doppler ultrasonography. All PICC insertions were performed under ultrasound guidance, and the right catheter tip position (at the junction of the superior vena cava and right atrium) was confirmed by X-ray.

The exclusion criteria were as follows: (1) existing venous thrombotic lesions before catheter placement; (2) ongoing anticoagulation therapy; (3) a history of central venous catheter insertion; (4) unplanned catheter removal caused by mechanical complications (e.g., catheter dislodgement or fracture); (5) incomplete clinical data or missing laboratory parameters required for analysis; and (6) inability to complete the follow-up of catheter treatment outcomes.

### 2.2 CRT screening and diagnosis

Following implantation of the PICC, patients received routine portable ultrasound examination of veins, such as the noble veins, median cubital veins, cephalic veins, axillary veins, and subclavian veins, with a specific focus on deep veins of the upper limb on the catheterization side, to reveal the presence of any catheter tip malpositioning or thrombosis. During follow-up, ultrasound was performed when patients developed CRT-related symptoms (e.g., pain, swelling, catheter dysfunction) or at the time of catheter removal. During follow-up, ultrasound was performed when patients developed CRT-related symptoms (e.g., pain, swelling, catheter dysfunction) or at the time of catheter removal.

CRT was confirmed by color Doppler ultrasound. All ultrasound examinations were performed by experienced ultrasound physicians. All the ultrasound images were taken by two experts. The diagnosis of CRT was established when at least two of the following color Doppler ultrasound criteria were met: (1) visualization of an intraluminal thrombus; (2) non-compressibility of the affected vein; (3) absence of venous flow on Doppler or color ultrasound; and (4) loss of phasicity of flow with respiration. These criteria are consistent with established diagnostic standards for catheter-related thrombosis (18, 19).

On the basis of clinical presentation, confirmed CRT cases were further classified into four categories: (1) deep vein thrombosis (DVT), possibly with elevated skin temperature and an ultrasound-confirmed thrombus in the corresponding vessel; (3) asymptomatic thrombosis, which is characterized by the absence of both subjective symptoms and objective abnormal signs; and (4) thrombotic catheter dysfunction, which is characterized by partial or complete infusion blockage caused by fibrin sheath or thrombus formation (20).

### 2.3 Patient demographics

Demographic and clinical data, including age, sex, body mass index (BMI), body temperature, heart rate, respiratory rate, systolic blood pressure, diastolic blood pressure, smoking history, alcohol abuse history, baseline comorbidities (hypertension history, diabetes history, chronic liver disease, and chronic kidney disease), thromboembolism history, blood transfusion history, use of mechanical ventilation, types of malignancy (DSTs and GTs), distant tumor metastasis, and laboratory parameters obtained after diagnosis, were retrospectively collected. Routine laboratory indicators included WBC count, platelet count, D-dimer, hemoglobin, and hs-CRP levels. All variables were collected from the electronic medical records by trained researchers using a standardized case report form. Only patients with complete data for all targeted variables were included in the final analysis; accordingly, no missing data imputation was needed.

### 2.4 Statistical analysis

All analysis were performed on the complete dataset (n=270) with no missing values for any variables included in the study. All the statistical analysis were performed using SPSS 25.0 and R software (version 4.2). Continuous variables were first assessed for normality; normally distributed data are presented as the mean ± standard deviation and were compared using independent or paired-sample t-tests, whereas non-normally distributed data are summarized as medians with interquartile ranges and were compared using the Mann–Whitney U test. Categorical variables are expressed as frequencies and percentages, with group comparisons performed using the χ² test or Fisher’s exact test, as appropriate. For multivariable analysis, variables with P < 0.05 in univariate analysis were entered into a binary logistic regression model using a stepwise backward selection method (likelihood ratio test) to identify independent predictors of CRT. The discriminative performance of the final model was evaluated using the area under the curve (AUC), with calculations of the optimal cutoff value, sensitivity, and specificity. Calibration was assessed via the Hosmer–Lemeshow test and calibration curves, while decision curve analysis was used to evaluate the clinical net benefit across a range of threshold probabilities. To mitigate potential over-optimism stemming from the relatively small number of CRT events, we performed 1,000 bootstrap resampling iterations with replacement within the training cohort. For each iteration, the model was re-estimated and then evaluated on both the resampled subset and the complete original training data; the discrepancy in the C-statistic between these two evaluations served as the optimism estimate for that iteration. The mean optimism across all iterations was subsequently deducted from the apparent C-statistic, yielding the optimism-adjusted C-statistic. Furthermore, coefficient stability was assessed by deriving 95% confidence intervals for each regression parameter via the bootstrap percentile method. Finally, a nomogram was developed from the ultimate multivariable model to serve as a graphical instrument for individualized CRT risk prediction.

## 3 Results

### 3.1 Baseline Data

In this study, 270 patients who underwent treatment for malignant tumors were included. The group characteristics are described in **Table 1**. There were 120 patients with DSTs and 150 patients with GTs. Symptomatic CRT occurred in 46 patients (17%), including 26 (56.5%) with DSTs and 20 (43.5%) with GTs. The median age of the CRT group was 73 years, with 56.5% being female. The non-CRT group (N = 224) had a median age of 71 years, with 52.7% being female, and the sex composition did not significantly differ between the two groups (*p >*0.05). In terms of clinical history, compared with the non-CRT group, the CRT group had a significantly greater heart rate (101.4 vs. 96.1; *p* < 0.01), history of diabetes rate (67.4% vs. 29%; *p* < 0.001), history of thromboembolism rate (50% vs. 16.1%; *p* < 0.001), mechanical ventilation usage rate (42.8% vs. 20.1%; *p* < 0.001), and incidence of distant cancer metastasis rate (37% vs. 4.9%; *p* < 0.001). Laboratory indicators revealed that the CRT group had significantly elevated WBC cunts (11.1 vs. 8.9×10^9^/L; *p* < 0.001), D-dimer levels (16.0 mg/L vs. 6.1 mg/L; *p* < 0.001), and hs-CRP levels (15.8 mg/L vs. 5.2 mg/L; *p* < 0.001), while the platelet count was significantly lower (126.9 vs. 152.7; *p* < 0.001). BMI, body temperature, respiratory rate, rates of smoking history, alcohol abuse history, hypertension, chronic liver disease, chronic kidney disease, and distribution of tumor types were comparable and did not significantly differ between the two groups (*p >*0.05).

**Table 1:** Demographic and clinical characteristics of the study population at baseline.

Characteristic	CRT (N = 46)	non-CRT (N = 224)	t/χ2	*P* Value
Age(yr)	73 (68, 78)	71 (65, 75)	2.268	0.024
Female sex	26 (56.5%)	118 (52.7%)	0.226	0.634
Body mass index (kg/m^2^)	24.9 (22.4, 27)	25 (22.5, 27.5)	-0.114	0.914
Temperature (°C)	36.8 (36.4, 37.1)	36.8 (36.4, 37.2)	-0.221	0.826
Heart rate (beats/min)	101.4 (89.8, 114)	96.1 (86, 105.8)	2.693	0.008
Respiratory rate (breaths/min)	18.2 (17, 20)	18.5 (17, 20)	-0.881	0.382
Systolic blood pressure (mmHg)	135.8 (123, 150)	131.1 (121, 141)	1.834	0.071
Diastolic blood pressure (mmHg)	84.7 (77,92)	81.5 (74, 89)	1.982	0.051
Smoking	12 (26.1%)	48 (21.4%)	0.479	0.489
Drinking	12 (26.1%)	51 (22.8%)	0.235	0.628
Hypertension	24 (52.2%)	94 (41.2%)	1.617	0.204
Diabetes	31 (67.4%)	65 (29%)	24.525	<0.001
Chronic liver disease	9 (19.6%)	36 (16.1%)	0.335	0.562
Chronic kidney disease	4 (8.7%)	12 (5.4%)	0.763	0.382
History of thromboembolism	23 (50%)	36 (16.1%)	25.726	<0.001
History of blood transfusion	8 (17.4%)	36 (16.1%)	0.049	0.825
Mechanical Ventilation	22 (42.8%)	45 (20.1%)	15.737	<0.001
Digestive system tumors	26 (56.5%)	124 (55.4%)	0.021	0.885
Gynecologic tumors	20 (43.5%)	100 (44.6%)	0.021	0.885
Distant metastasis	17 (37%)	11 (4.9%)	42.164	<0.001
WBC count (10^9^/L)	11.1 (9.4, 10.6)	8.9 (6.9, 10.6)	5.763	<0.001
Platelet count (10^9^/L)	126.9 (96, 146.3)	152.7 (139, 169.8)	-5.533	<0.001
D-dimer (0.1mg/L)	16.0 (11.6, 22.8)	6.1 (4.2, 8.2)	11.29	<0.001
Hemoglobin (g/L)	95.7 (80.8, 108.6)	97.1 (83.4, 110.4)	-0.54	0.65
hs-CRP (mg/L)	15.8 (8.9, 22.1)	5.2 (2.1, 6.1)	10.81	<0.001

### 3.2 Risk factors for CRT in patients with DSTs or GTs

On the basis of the results of univariate analysis, CRT occurrence was used as the dependent variable, with age, proportion of diabetes, history of thromboembolism, heart rate, mechanical ventilation usage rate, distant metastasis of cancer, WBC count, platelet count, D-dimer level, and hs-CRP level as independent variables for multivariate logistic regression analysis (**Table 2**). A history of thromboembolism (OR = 6.371, 95% CI = 1.067–38.034), use of mechanical ventilation (OR = 6.081, 95% CI = 1.077–34.345), distant metastasis of cancer (OR = 23.791, 95% CI = 2.037–277.883), high WBC count (OR = 1.646, 95% CI = 1.165–2.327), D-dimer level (OR = 1.733, 95% CI = 1.339–2.242), high-sensitivity C-reactive protein (hs-CRP) level (OR = 1.207, 95% CI = 1.087–1.341), and low platelet count (OR = 0.954, 95% CI = 0.927–0.982) were independent predictors of CRT occurrence (**Table 2**).

**Table 2:** Multivariate logistic regression analysis of independent risk factors for CRT formation.

Parameters	ß	SE of ß	OR	95% CI	Wald value	*P* value
Age (years)	0.042	0.064	1.043	0.921-1.182	0.441	0.507
Diabetes	0.666	0.878	1.947	0.348-10.880	0.576	0.448
History of thromboembolism	1.852	0.912	6.371	1.067-38.034	4.127	0.042
Heart rate (beats/min)	0.031	0.033	1.031	0.967-1.099	0.874	0.350
Mechanical Ventilation	1.805	0.883	6.081	1.077-34.345	4.177	0.041
Distant metastasis	3.169	1.254	23.791	2.037-277.883	6.387	0.011
WBC (10^9^/L)	0.499	0.177	1.646	1.165-2.327	7.978	0.005
Platelet (10^9^/L)	-0.047	0.015	0.954	0.927-0.982	10.225	0.001
D-dimer (0.1mg/L)	0.550	0.132	1.733	1.339-2.242	17.459	<0.001
hs-CRP (mg/L)	0.189	0.054	1.207	1.087-1.341	12.392	<0.001

ß, regression coefficient; CI, confidence interval for odds ratio; OR, odds ratio; SE, standard error; WBC, white blood cell; hs-CRP, high-sensitivity C-reactive protein.

### 3.3 Construction of the predictive model

Five risk factors were retained in the model (p < 0.015). However, given the limited number of CRT events (n=46), we further assessed coefficient stability using 1,000 bootstrap resamples. This revealed that distant metastasis had a bootstrap 95% CI crossing zero (-0.252 to 467.013), indicating instability. Therefore, we removed this variable and fitted the model with four stable laboratory markers (WBC, platelet count, D-dimer level, and CRP level). The final model had an events per variable (EPV) of 46/4 = 11.5, which exceeded the recommended threshold of 10, and all four coefficients demonstrated stable bootstrap 95% CIs that did not cross zero.

**Figure 1:**
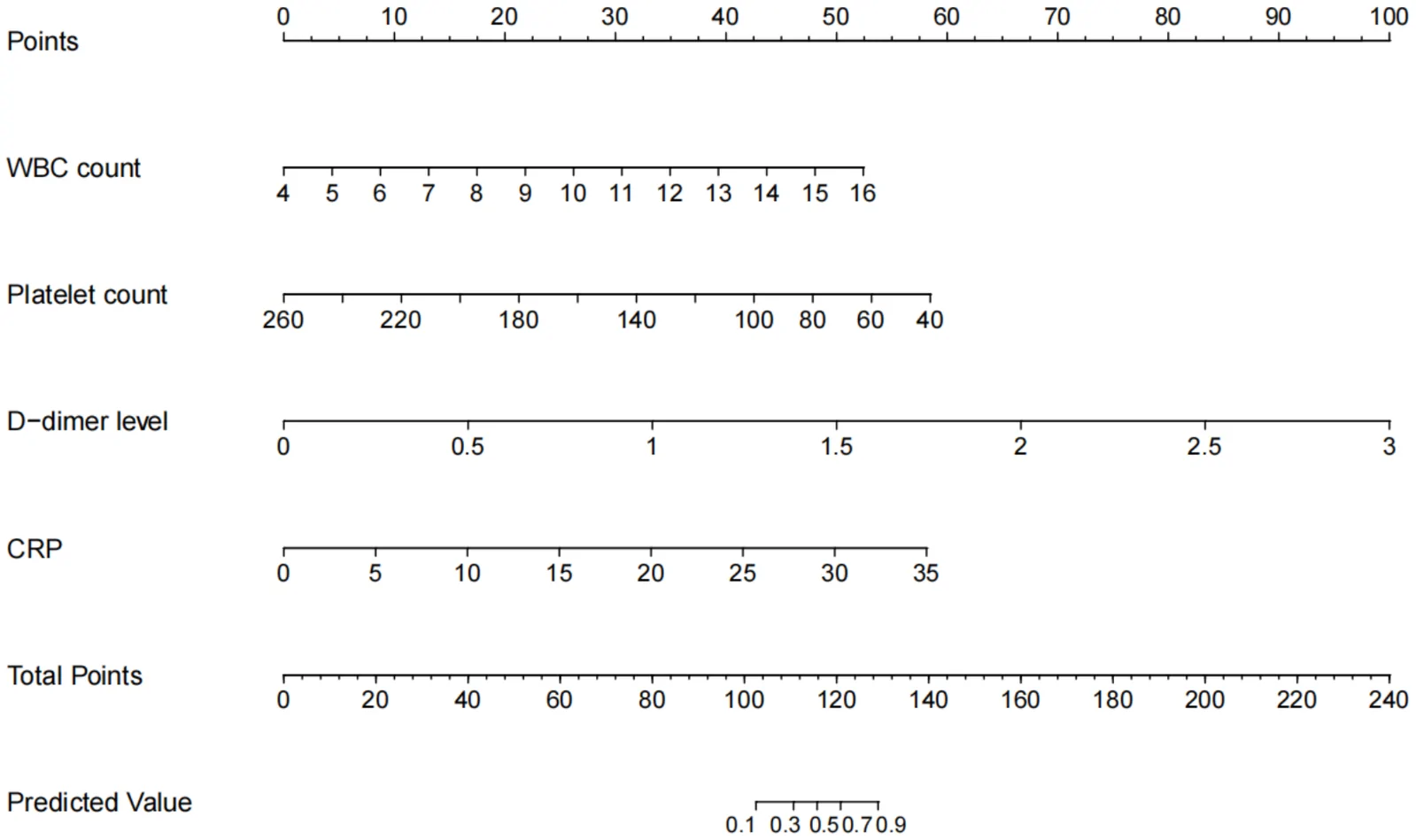
Nomogram for predicting CRT occurrence in cancer patients.

A nomogram was constructed on the basis of the final 4-variable model to visualize individual CRT risk. In this nomogram, a total score of 100 points corresponded to a detectable thrombotic risk, and a score of 130 points substantially increased the probability of CRT (**Figure 1**).

### 3.4 Model evaluation and validation

**Figure 2:**
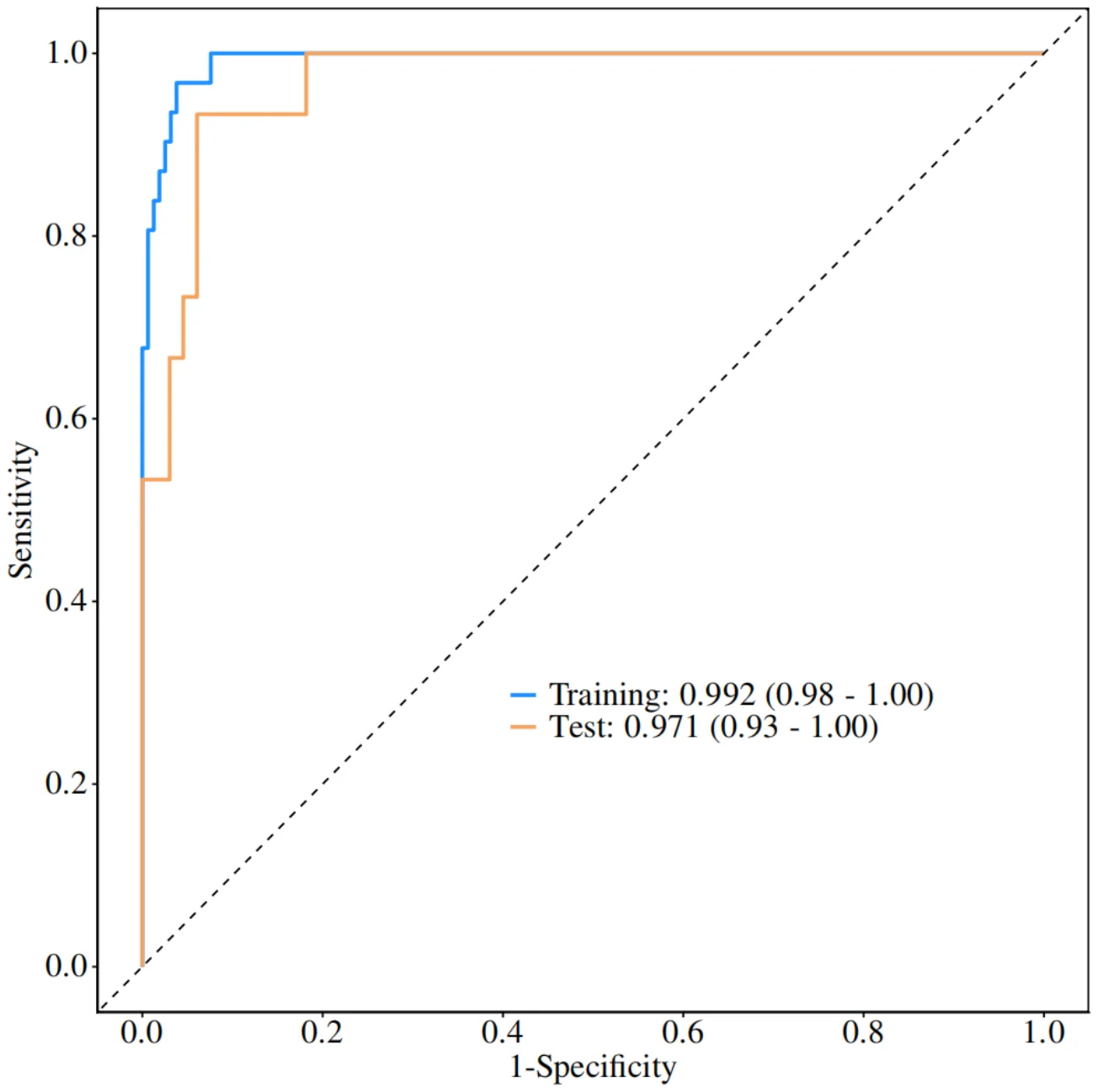
ROC curves of the model training group and the test group.

**Figure 3:**
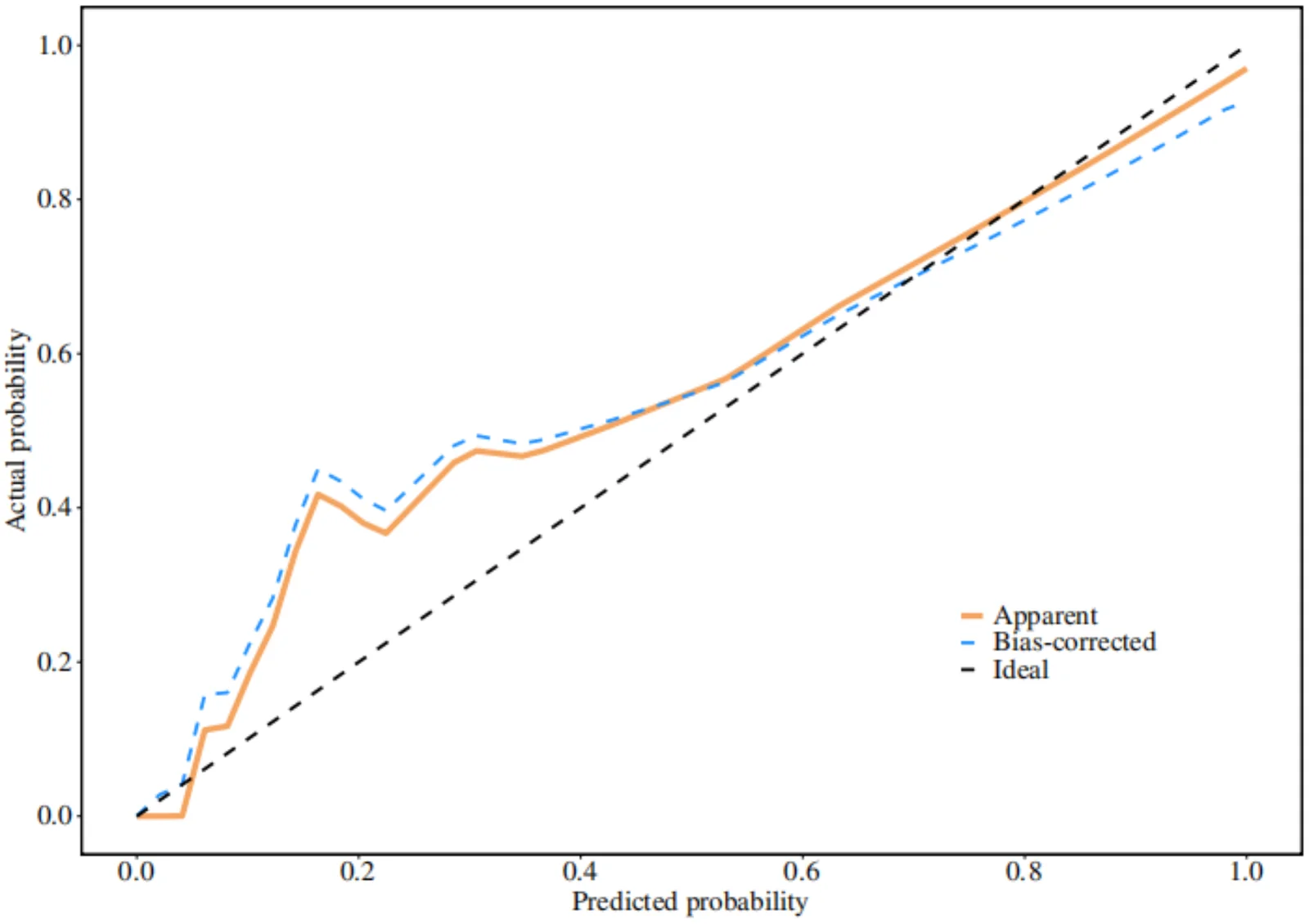
Calibration curve. The 45° diagonal line in the figure represents the ideal curve (Ideal), the solid line in the figure is the original curve (Apparent), and the dashed line represents the calibrated curve (Bias-corrected). If the predicted calibration curve is closer to the ideal curve, the model's accuracy is better.

The final 4-variable model achieved an apparent C-statistic of 0.992 in the training cohort. After 1,000 bootstrap resamples with optimism correction, the bias-corrected C statistic remained high at 0.986 (mean optimism = 0.006), indicating minimal overfitting. In the independent test set, the model yielded an AUC of 0.971 (**Figure 2**; **Table 3**). Internal validation was performed using the bootstrap method with 1,000 resamplings. The results of the calibration curve test were p =0.9933, indicating the good calibration capability of the model (**Figure 3**).

**Table 3:** Diagnostic value analysis of the training model.

AUC	95% CI	Cut-off	Sensitivity (%)	Specificity (%)	Youden Index	*p* value
0.992	0.98-1.00	0.12	100%	90%	0.90	<0.001

AUC, area under the curve; CI, confidence interval for odds ratio.

### 3.5 Clinical utility evaluation

**Figure 4:**
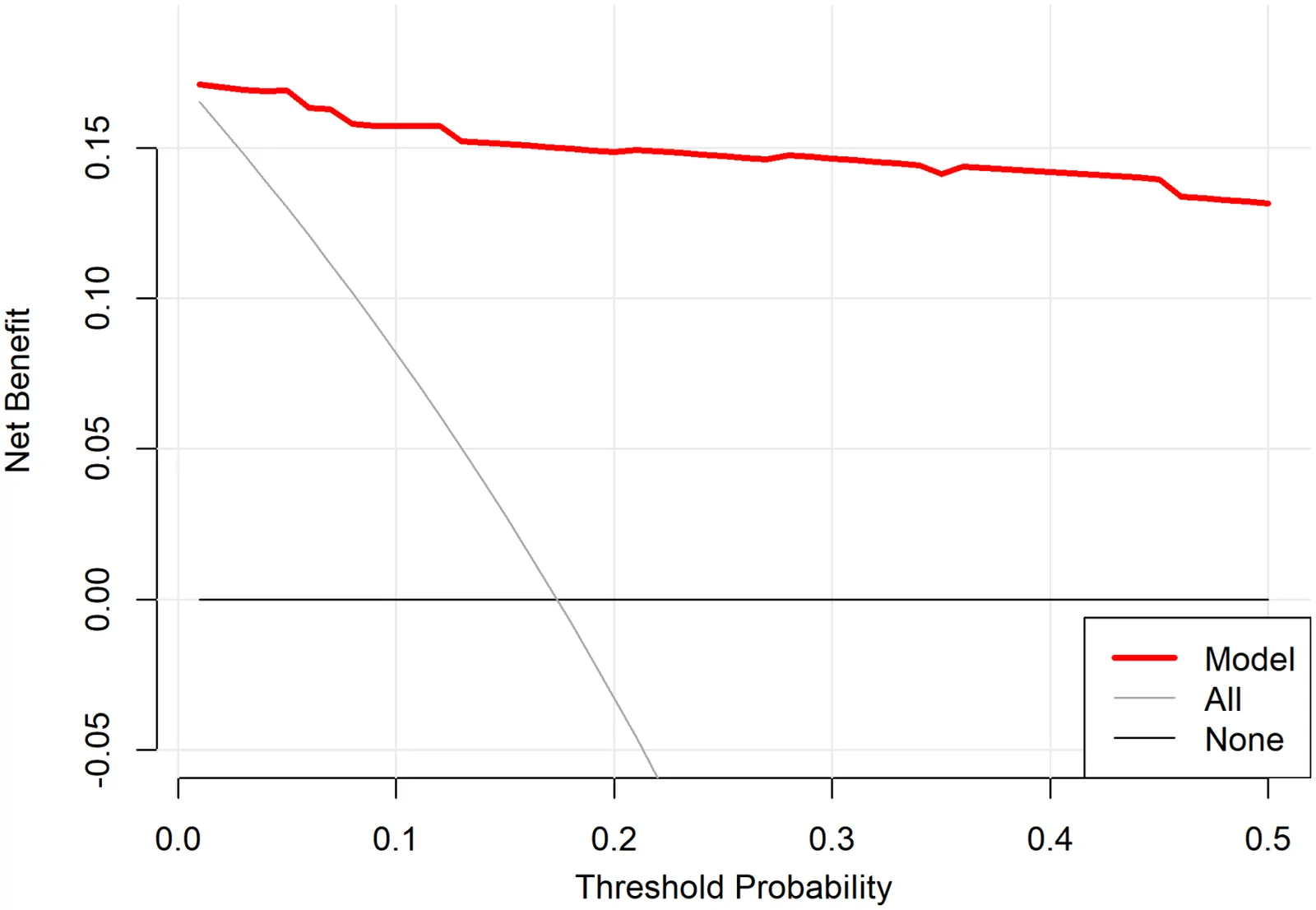
Clinical decision curve of the model.

To evaluate the clinical net benefit of the model under different threshold probabilities, clinical decision curve analysis (DCA) was further employed to test its clinical applicability. The results showed that the model has a wide range of threshold probabilities and high potential for clinical application, with its net benefit level significantly higher than that of the “no intervention” strategy (nonline) and the “intervention for all” strategy (all line), indicating that the model has value for clinical application. The optimal cutoff value of 0.12 determined by ROC analysis falls precisely within the effective threshold range of the DCA curve, further validating the clinical effectiveness of this predictive model (**Figure 4**).

## 4 Discussion

In this study, we developed and internally validated a CRT risk prediction model for elderly patients with DSTs or GTs. Four variables, namely, WBC count, platelet count, D-dimer level, and hs-CRP level, were used to construct the model, which demonstrated good discriminative ability (optimism-corrected AUC = 0.986) and clinical utility.

The widespread use of CVCs is associated with various complications, including acute events such as pneumothorax, inadvertent arterial puncture, and hematoma, as well as long-term complications such as CRT and catheter-related infections (21, 22). A recent meta-analysis of 30 studies involving ICU patients demonstrated that invasive mechanical ventilation is significantly associated with an increased risk of venous thromboembolism (VTE) (OR = 2.08, 95% CI: 1.46–2.96) (23). In critically ill patients with tumors, mechanical ventilation has been incorporated as a component of the ICU VTE score (2 points), further supporting its role as a clinically relevant predictor in this specific population (24). The prothrombotic effects of mechanical ventilation are mediated through multiple interconnected pathways. First, the mechanical stress generated during positive-pressure ventilation induces a systemic inflammatory response characterized by elevated levels of pro-thrombotic cytokines such as IL-1β, IL-6, IL-8, and TNF-α (25, 26). Second, mechanical ventilation has been shown to cause systemic elevation of thrombin-antithrombin complexes, indicating ongoing activation of the coagulation cascade (27). Third, patients receiving mechanical ventilation often require sedation and neuromuscular blockade, leading to prolonged immobility and venous stasis, which further exacerbates thrombotic risk (28). Elderly patients often have multiple underlying conditions (e.g., diabetes), which can further exacerbate the risk of thrombosis (29). In our cohort, the incidence of CRT was 17.04%. 67.4% of patients with CRT had comorbid diabetes, while only 29% of patients without CRT had comorbid diabetes. Additionally, we reported that 42.8% of patients in the CRT group received mechanical ventilation, which was significantly greater than that in the non-CRT group. For critically ill patients at high risk of bleeding, clinical guidelines recommend thromboprophylaxis solely through mechanical means, as anticoagulant use is contraindicated in such cases (30). Mechanical ventilation, a critical supportive measure to alleviate respiratory failure symptoms and optimize oxygen delivery to tissues and vital organs, can effectively maintain metabolic demands and preserve vital organ function during acute illness. These patients often require central venous access to meet clinical needs, such as acute resuscitation, frequent laboratory testing, hemodynamic monitoring, and safe medication administration (31).

Under physiological conditions, blood flows in a laminar state during normal circulation. This laminar characteristic allows blood components to form specific distributions within blood vessels, maintaining a certain blood flow velocity and volume, which serves as the hemodynamic basis for antithrombotic mechanisms under normal physiological conditions (32). In cancer patients, malignant cells can secrete tissue factor and other procoagulant molecules. These molecules directly interfere with the normal activation processes of platelets, white blood cells, and endothelial cells through direct cell-to-cell contact or by inducing the production of proinflammatory cytokines. Overall, cancer-related biological processes disrupt the normal coagulation cascade and physiological fibrinolysis system balance, often leading to pathological local and systemic hypercoagulable states (33, 34). Thrombosis risk varies due to different tumor types, stages, histological grades, and tumor genomic profiles, especially for tumors with aggressive biological behavior and clinical features, as well as in advanced malignant diseases (35).

The biological mechanisms linking malignancy, inflammation, and CRT are multifaceted. Cancer-derived tissue factor and procoagulant molecules directly activate the extrinsic coagulation cascade, while tumor-associated inflammation drives the release of proinflammatory cytokines (e.g., IL-1β, IL-6, TNF-α), which further promote a prothrombotic state through endothelial activation, upregulation of tissue factor expression, and platelet activation (36). Siniscalchi et al. (37) suggest that statins produce anticoagulant effects via the downregulation of tissue factor expression and increased endothelial thrombomodulin expression, resulting in reduced thrombin generation. They highlight the central role of these pathways in thrombus formation. In our cohort, the significantly elevated WBC count and hs-CRP levels in CRT patients indicate a relatively higher systemic inflammatory state, whereas the elevated D-dimer level directly indicates ongoing fibrin turnover and hypercoagulability. Together, these findings support the notion that CRT in cancer patients is driven by a convergence of inflammation, hypercoagulability, and endothelial dysfunction—which is consistent with the classical Virchow’s triad.

An elevated white blood cell count is closely associated with an increased risk of thrombosis. A prospective study involving 2510 cancer patients confirmed that when the white blood cell count exceeded 11 × 10^9^/L, the risk of thrombosis significantly increased (38). White blood cells promote the activation of the coagulation system through the secretion of inflammatory mediators (39). Additionally, PSGL-1 on the surface of white blood cells can specifically bind to P-selectin receptors on the surface of platelets, resulting in the formation of leukocyte–platelet aggregates (PLAs). These aggregates further promote thrombosis by enhancing platelet activation, adhesion, and aggregation (40). Consistent with previous reports, we found that the median WBC count in the CRT group was 11.1 × 10^9^/L, which was significantly greater than that in the non-CRT group, suggesting that leukocytosis may promote thrombosis through inflammatory pathways.

In addition to white blood cells, high D-dimer levels are also associated with the development of CRT. D-dimer, as a soluble fibrin degradation product, originates from the degradation of cross-linked fibrin by plasmin and can significantly increase or result in positive reactions in secondary hyperfibrinolysis states (such as hypercoagulability, disseminated intravascular coagulation, and thrombolytic therapy) (41, 42). Elevated D-dimer levels suggest the possible presence of thrombotic diseases from multiple sources and enhanced fibrinolytic activity. In cancer patients, high D-dimer concentrations can serve as monitoring indicators following tumor resection, for cancer thrombosis, and after intravenous chemotherapy. This study confirms that elevated D-dimer is an independent risk factor for CRT. Song et al. (43) reported that D-dimer levels are significantly increased in cancer patients. D-dimer testing also has high specificity for the diagnosis of VTE during pregnancy (44). To identify and reduce the incidence of CRT early, dynamic monitoring of D-dimer levels is necessary during the perioperative period. Notably, Thmo et al. (45) reported that CRT is more often symptomatic, whereas TIVAP-associated CRT tends to be asymptomatic. For patients with PICC catheters, routine vascular ultrasound examination is needed to confirm the presence of thrombus when CRT-related symptoms such as pain, swelling, elevated skin temperature, or catheter dysfunction occur. For TIVAP patients, even in the absence of typical symptoms, a combined strategy of D-dimer level monitoring and ultrasound screening can be considered to optimize thrombus prevention and intervention timing.

Beyond being a mechanical complication of catheter placement, CRT may represent a manifestation of an underlying prothrombotic phenotype in cancer patients. VTE has been consistently associated with increased mortality and worse overall prognosis in patients with malignant tumors. In a meta-analysis including 873,292 records, Yang et al. (46) demonstrated that VTE occurrence is significantly influenced by tumor stages and node and metastasis status, as well as chemotherapy. In the most recent meta-analysis enrolling 96,753 patients, multiple factors were found to be associated with increased risk of recurrent VTE, which include advanced cancer, previous history of VTE, and specific cancer types (eg. lung cancer, pancreas cancer, hepatobiliary carcinoma, and genitourinary) (47).

For early identification of CRT in patients with cancer, several prediction models for CRT have been developed. Wang et al. (48) developed a nomogram for CRT prediction in 4,691 cancer patients with CVC placement (nine predictors, AUC 0.832). Lin et al. (49) developed a nomogram for 647 cancer patients (four predictors, including D-dimer concentration; AUC, 0.757). Wang et al. (50) developed a nomogram for PICC-related thrombosis in 305 lymphoma patients (five predictors, AUC 0.907). Taglialatela et al. (10) evaluated existing risk scores (Khorana, Protecht, COMPASS-CAT, and Michigan) in oncological patients and reported significant associations with high-grade scores. Compared with these models, our nomogram demonstrated greater discriminative ability (optimism-corrected AUC = 0.986; test-set AUC = 0.97) and greater parsimony (four predictors). D-dimer consistently emerged as a predictor across studies, supporting its central role in CRT pathogenesis. However, direct comparisons should be interpreted with caution, as these studies differed in patient population, catheter type, sample size, and study design. External validation in independent cohorts is still warranted.

## 5 Limitations

This study has several limitations. This study has several limitations. First, as this was a retrospective single-center study with a relatively small sample size (270 patients), the findings are subject to selection and information biases, and the strict exclusion criteria may have further introduced selection bias. Second, variable selection was driven primarily by univariate screening and clinical plausibility, and we did not explore potential nonlinear relationships. Third, while we performed 1,000 bootstrap resamples for optimism correction and used an independent test set from the same institution, these approaches cannot replace external validation in a distinct patient cohort, and the model’s performance may be overestimated because of shared center-specific characteristics. Therefore, our results should be considered exploratory and preliminary. We do not recommend clinical application unless the model has been validated in larger, multicenter prospective cohorts. Future work should prioritize external validation across diverse populations and incorporate systematic ultrasound screening protocols to more accurately capture the full spectrum of CRT events, including asymptomatic cases.

## 6 Conclusion

Elevated WBC count, D-dimer level, and hs-CRP level and decreased platelet count were identified as independent risk factors for CRT in elderly hospitalized cancer patients. The predictive model based on these four variables demonstrated good discriminative ability in internal validation and a single-center test set. However, external validation in larger, multicenter prospective cohorts is needed before the model can be considered for clinical application.

## Data Availability

The original contributions presented in the study are included in the article/supplementary material. Further inquiries can be directed to the corresponding authors.
